# Impact of Lifestyle on Overall Cancer Risk among Japanese: The Japan Public Health Center-Based Prospective Study (JPHC Study)

**DOI:** 10.2188/jea.JE20090209

**Published:** 2010-03-05

**Authors:** Manami Inoue

**Affiliations:** Epidemiology and Prevention Division, Research Center for Cancer Prevention and Screening, National Cancer Center, Tokyo, Japan

**Keywords:** cancer, risk factor, attributable fraction, Japanese, cohort study

## Abstract

In Japan, cancer has long been recognized as a major component of the overall pattern of disease. Currently, there is a need to implement practical control measures with specific numerical targets appropriate for the Japanese population. Using data from the Japan Public Health Center-based Prospective Study, the author estimated the impact of major risk factors on overall cancer risk among a Japanese population. These risk factors included tobacco smoking, alcohol drinking, body mass index, history of diabetes, physical activity, and metabolic factors and their aggregates. The results show that tobacco smoking and heavy alcohol drinking were significantly positively associated with overall cancer risk, and that total physical activity was significantly inversely associated with the risk of cancer. Although people with a history of diabetes may be at increased risk of cancer, extreme body mass index and metabolic factors in the aggregate had little impact on overall cancer risk in the Japanese population.

## INTRODUCTION

In Japan, cancer has been recognized as a major component of the overall pattern of disease for decades. Thus, the importance of cancer prevention through lifestyle modification is now widely acknowledged. Internationally, several studies have used epidemiologic evidence to estimate the proportion of all cancers attributable to a number of risk factors, and various international guidelines and recommendations derived from these have been promulgated.^1^^,^^2^ Not surprisingly, Japanese domestic guidelines and recommendations for cancer prevention have been significantly influenced by these reports. The current need is to implement practical control measures with specific numerical targets appropriate for the Japanese population. Sufficient and reliable data derived from the Japanese population are therefore needed. Estimation of the expected effectiveness of primary prevention requires calculation of the fraction of the population incidence rate of a cancer that can be attributed to major risk factor. However, there are limited data on major risk factors and subsequent cancer risk in Japan.

We launched a large-scale, population-based, prospective study in 1990 in 11 public health center-based areas throughout Japan. The subjects were 140 420 middle-aged residents, and information was collected by using questionnaire surveys, blood samples, health screening data, and a thorough follow-up system.^3^ The follow-up period is currently 10 to 15 years and a sufficient number of incident cancers has accumulated. Here, to develop a relevant epidemiological index of the impact of major risk factors—tobacco smoking, alcohol drinking, body mass index (BMI), history of diabetes mellitus (DM), physical activity, and metabolic factors and their aggregates—on overall cancer risk among the Japanese general population, we conducted cohort analyses using data from the Japan Public Health Center-based Prospective Study (JPHC study).

## THE JPHC STUDY

The JPHC Study was launched in 1990 for Cohort I and in 1993 for Cohort II. Cohort I comprised 5 prefectural public health center (PHC) areas: Ninohe (Iwate Prefecture), Yokote (Akita Prefecture), Saku (Nagano Prefecture), Chubu (Okinawa Prefecture), and Katsushika (metropolitan Tokyo). Cohort II comprised 6 PHC areas: Mito (Ibaraki Prefecture), Nagaoka (Niigata Prefecture), Chuo-higashi (Kochi Prefecture), Kamigoto (Nagasaki Prefecture), Miyako (Okinawa Prefecture), and Suita (Osaka Prefecture). Details of the study design are described elsewhere.^3^^,^^4^ The study population was defined as all registered Japanese inhabitants aged 40 to 59 years (for Cohort I) or 40 to 69 years (for Cohort II) at baseline. Participants were identified by resident registries maintained by local municipalities. This study was approved by the institutional review board of the National Cancer Center of Japan. In the present series of analyses, the Katsushika PHC area was excluded because data on cancer incidence were not available.

A baseline self-administered questionnaire survey was conducted in 1990 to 1994, and a 5-year follow-up questionnaire in 1995 to 1999, with a response rate of around 80%. Subjects with a history of cancer at any site were excluded from the analysis.

Subjects were followed from the starting point until the end of follow-up, which depended on the particular analysis. Residence status, including survival, was confirmed through the residential registry. Access to the resident registry is available to anyone, as mandated by the resident registration law. Among the study subjects, approximately 0.5% were lost to follow-up during the follow-up period. Information on the cause of death for deceased subjects was obtained from death certificates (provided by the Ministry of Health, Labour, and Welfare, with the permission of the Ministry of Internal Affairs and Communications), on which cause of death is classified according to the International Classification of Diseases, Tenth Revision.^5^ Resident registration and death registration are required by law in Japan, and the registries are believed to be complete. Incident cancers were identified via notification from the major hospitals in the study area and by data linkage with population-based cancer registries. Death certificates were used as a supplementary information source. The site and histology of each cancer were coded using the International Classification of Diseases for Oncology, Third Edition.^6^ In our cancer registry system, the proportion of cases for which information was available from death certificates only (DCO) was around 4%.

Hazard ratios (HRs) and their 95% confidence intervals (95% CIs) were used to describe the relative risk of overall cancer occurrence associated with the presence of major risk factors at the start of each study. The Cox proportional hazards model was used for the analysis, after controlling for potential confounding factors in addition to age and study area.

To express the impact of major risk factors on overall cancer occurrence in this population, the population attributable fraction (PAF) was estimated. This is the fraction of the population incidence rate of cancer that can be attributed to a particular cause—in other words, the reduction in incidence that would be expected had the population been entirely unexposed.^7^ PAF was estimated as:

$$\mathrm{pd}\times \left(\mathrm{HR}-\frac{1}{\mathrm{HR}}\right)$$

where **pd** is the proportion of cases exposed to a particular risk factor. This formula is believed to have greater validity, when confounding variables are present, than the more common formula:

$$\frac{\mathrm{Pe}(\mathrm{HR}-1)}{\mathrm{Pe}(\text{HR}-1)+1}$$

where **Pe** is the proportion of the source population exposed to a particular risk factor.^8^ We used the formula of Greenland to estimate the 95% CIs of adjusted PAFs.^9^

### Impact of tobacco smoking on subsequent cancer risk (Figure 1)^10^

Although the relations between tobacco smoking and cancers at various sites are unequivocal, few studies have examined the subsequent risk and PAF of overall cancer incidence in relation to tobacco smoking. This study aimed to develop a relevant epidemiological index of the impact of tobacco smoking on the subsequent risk of cancer in Japan. We conducted a cohort analysis of the possible association between tobacco smoking habits and overall cancer risk among 92 792 subjects (44 521 men and 48 271 women), with a follow-up period of 9.6 years. From 1990 through 2001, there were 4922 incident cases of cancer (2969 men and 1953 women). Responses to the baseline questionnaire indicated that 52.2% of men and 5.6% of women were current smokers, among whom the HR for subsequent cancer occurrence, as compared with never smokers, was 1.64 (95% CI, 1.48–1.82) and 1.46 (1.21–1.75), respectively. The corresponding PAF of overall cancer incidence in men was 22.4% (15.7%–28.5%) and 7.0% (3.7%–10.3%) in relation to current and past exposures to tobacco smoke. In women, the respective PAFs were only 2.2% and 0.6%, due to the low prevalence of current and former smokers. Our results suggest that avoidance of tobacco smoking would prevent 29% of cancers in men and 3% of cancers in women.

**Figure 1.**
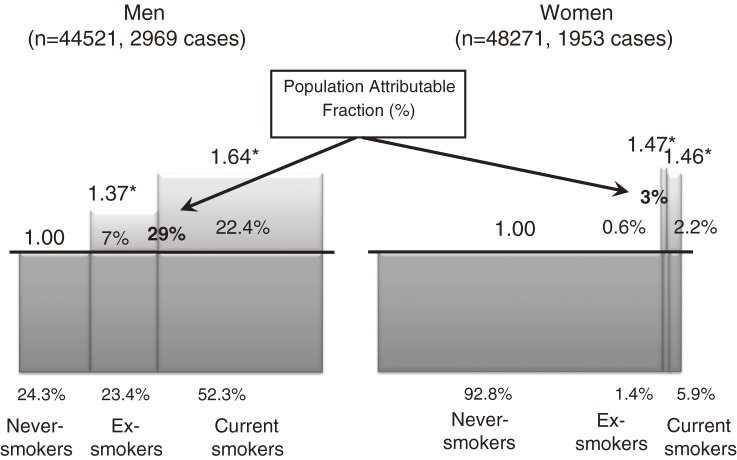
Impact of tobacco smoking on subsequent cancer risk in men and women^10^

### Impact of alcohol drinking on overall cancer risk (Figure 2)^11^

In Japan, both alcohol consumption and the proportion of heavy drinkers have been increasing for decades, and alcohol drinking has been recognized as an important and preventable public health problem. The epidemiological background, types of beverages regularly consumed, and genetic polymorphisms for alcohol-related enzymes in Japanese differ from those in Western populations. We conducted a cohort study of alcohol consumption and overall cancer incidence in 73 281 subjects (35 007 men and 38 274 women) aged 40 to 59 years at baseline over a 9.8-year follow-up period. During the period from 1990 through 2001, we identified a total of 3403 cases of newly diagnosed cancer and 1208 cancer deaths. In men, occasional drinkers had the lowest risk of developing cancer, and a positive linear association with ethanol intake was noted: the HRs were 1.18 (95% CI, 0.96–1.44) for 1 g to <150 g per week, 1.17 (0.96–1.44) for 150 g to <300 g per week, 1.43 (1.17–1.75) for 300 g to <450 g per week, and 1.61 (1.32–1.97) for 450 g or more per week (*P* for trend, <0.001). The positive association was more striking among current smokers and for alcohol-related cancers. Relatively few women were regular drinkers. Our results suggest that ethanol intake elevates the risk of cancer in a dose-dependent manner, and that nearly 13% of cancers among men in this study were due to heavy drinking (≥300 g per week of ethanol), to which smoking substantially contributed. Reduction of smoking is therefore important in decreasing the effect of alcohol on cancer risk.

**Figure 2.**
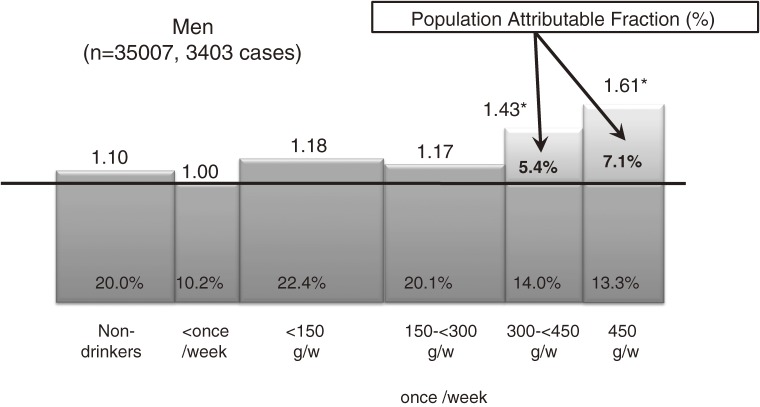
Impact of alcohol drinking on overall cancer risk in men^11^

### Impact of BMI on overall cancer risk (Figure 3)^12^

To determine whether BMI extremes in otherwise healthy individuals affect the likelihood that cancer will occur, we conducted a cohort analysis of the possible association between BMI and the risk of overall cancer incidence among 88 927 subjects (42 093 men and 46 834 women), with a 9.5-year follow-up. In men, there was a U-shaped association between BMI and cancer occurrence: men with a BMI of 23 to less than 25 had the lowest risk of cancer occurrence (BMI 14–<19: HR = 1.29, 95% CI = 1.08–1.54; BMI 30–<40: 1.22, 0.92–1.61). This tendency did not change substantially after excluding cases diagnosed early during the follow-up period; cancer mortality showed a similar trend, but with higher risk values. When analyzed according to smoking category, a low BMI had a stronger effect on cancer occurrence in current smokers than in never smokers. There was no marked fluctuation in risk in women. A very low BMI seems to have a greater impact on overall cancer risk in populations with a lower average BMI. Therefore, although much attention has been paid to the effects of obesity, the health effects at both BMI extremes should be considered in populations with a low average BMI.

**Figure 3.**
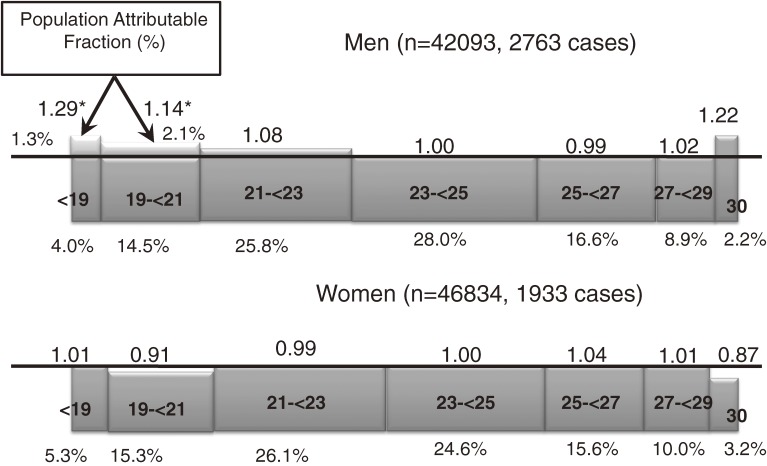
Impact of body mass index (BMI) on overall cancer risk^12^

### DM and the risk of cancer (Figure 4)^13^

As in many other countries, DM is a serious public health problem in Japan. One global estimate projects an increase in prevalence from 6.5% in 1995 to 8.7% in 2025 among Japanese aged 20 years or older. This increase in DM will likely influence trends in related health conditions, including cancer. Clarification of the association between DM and cancer in populations with an increasing DM prevalence, eg, Japanese, is thus a crucial task, not only with respect to causation but also with regard to the formulation of clinical strategies and public health policies for the target population.

**Figure 4.**
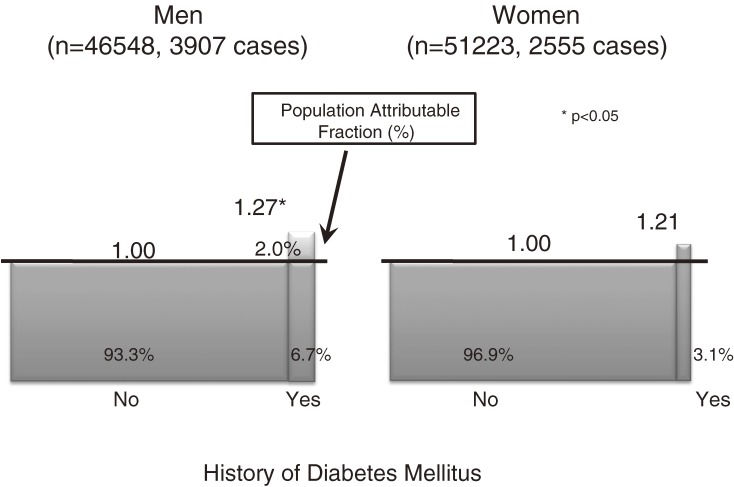
Diabetes mellitus and overall cancer risk^13^

We prospectively examined the association between a history of DM and subsequent risk of cancer. A total of 97 771 subjects (46 548 men and 51 223 women) who responded to the baseline questionnaire from 1990 through 1994 were followed up for cancer incidence through 2003. At baseline, 6.7% of men and 3.1% of women had a history of DM. A total of 6462 cases of newly diagnosed cancer were identified. In men, there was a 27% increase in the risk of overall cancer incidence in those with a history of DM (HR, 1.27; 95% CI, 1.14–1.42). The HRs were especially high for cancers of the liver (2.24, 1.64–3.04), pancreas (1.85, 1.07–3.20), and kidney (1.92, 1.06–3.46). We also observed a moderate increase in the risk of colon cancer (1.36, 1.00–1.85) and a borderline significant increase in stomach cancer (1.23, 0.98–1.54). In women, there were borderline significant increases in overall cancer risk (1.21, 0.99–1.47) and ovarian cancer (2.42, 0.96–6.09), and statistically significant increases in the risks for stomach cancer (1.61, 1.02–2.54) and liver cancer (1.94, 1.00–3.73). It appears that, among the general Japanese population, individuals with DM may be at increased risk for overall cancer and for cancer at specific sites.

### Daily total physical activity level and overall cancer risk (Figure 5)^14^

A number of investigators have reported that physical activity has beneficial effects on the risk of cancer at specific sites. As a result, physical activity is now regarded as an important target for cancer prevention. At present, however, information on the association between physical activity and overall cancer risk is limited. Given that exercise and physical activity probably affect cancer development at different sites via the same or very similar mechanisms, at least to some degree, it is reasonable to assess the preventive effect of physical activity not only on cancer at specific sites but also on all cancers in aggregate. Further, from a public health perspective, a better understanding of the preventive effect of physical activity on overall cancer risk would provide concrete information for estimating the effects of physical activity measures in health policy planning.

**Figure 5.**
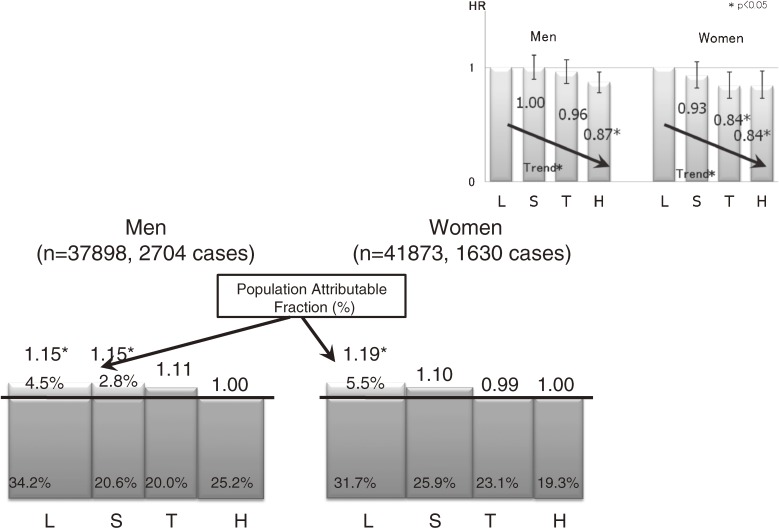
Daily total physical activity level and overall cancer risk^14^

We prospectively examined the association between daily total physical activity (using a score for metabolic equivalents per day) and subsequent cancer risk. A total of 79 771 Japanese men and women aged 45 to 74 years who responded to a questionnaire in 1995 through 1999 were followed for overall cancer incidence (4334 cases) through 2004. As compared with subjects in the lowest quartile, increased daily physical activity was associated with a significantly decreased risk of cancer in both sexes. In men, the HRs for the second, third, and highest quartiles were 1.00 (95% CI, 0.90–1.11), 0.96 (0.86–1.07), and 0.87 (0.78–0.96), respectively (*P* for trend = 0.005); in women, the HRs were 0.93 (0.82–1.05), 0.84 (0.73–0.96), and 0.84 (0.73–0.97), respectively (*P* for trend = 0.007). The decrease in risk was clearer in women than in men, especially among the elderly and those who regularly engaged in leisure sports or physical exercise. By site, decreased risks were observed for cancers of the colon, liver, and pancreas in men, and for cancer of the stomach in women. On estimation of the PAF from our results, 4.5% of male cases and 5.5% of female cases were considered preventable if the persons in the lowest physical activity category had increased their activity to a higher level. Increased daily physical activity may thus be beneficial in preventing cancer in a relatively lean population.

### Impact of metabolic factors on subsequent cancer risk (Figure 6)^15^

As in many countries, metabolic syndrome has recently attracted substantial attention in Japan, and this is reflected in the government’s decision to start a nationwide intervention strategy in April 2008. The National Health and Nutrition Survey in Japan reported that the prevalence of metabolic syndrome in the Japanese population aged 40 to 74 years in 2005 was 25.5% in men and 10.3% in women. Given the expectation that this would likely influence related health conditions, including cancer, clarification of the association between metabolic factors and cancer is a crucial task, not only with respect to causation, but also with regard to the formulation of clinical and public health strategies for the target population. However, the impact of metabolic factors on overall cancer risk has not been clarified.

**Figure 6.**
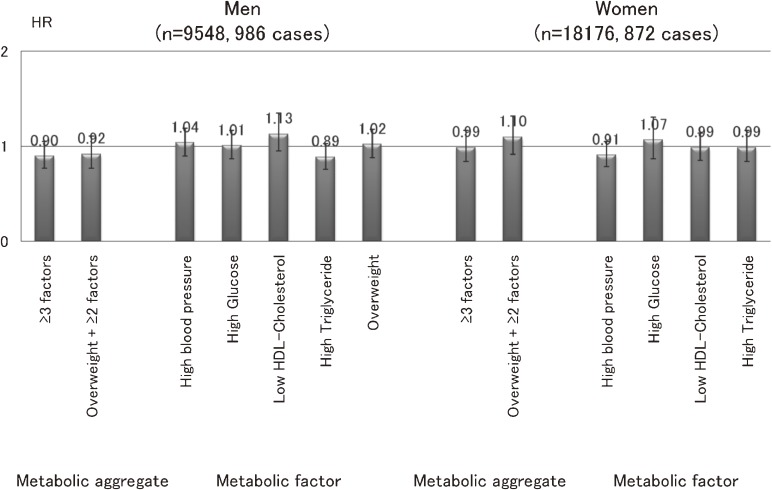
Impact of metabolic factors on subsequent cancer risk^15^

We prospectively examined whether metabolic factors and their aggregates predict the subsequent occurrence of overall and major sites of cancer. A total of 27 724 participants (9548 men and 18 176 women) aged 40 to 69 years who participated in a questionnaire and health checkup survey in 1993 through 1995 were followed for overall cancer incidence through 2004. HRs and 95% CIs were calculated for metabolic factors (hypertension, high serum glucose, low HDL-cholesterol, hypertriglyceridemia, and overweight) and for 2 aggregates of these criteria (≧3 factors; ≧2 additional factors, plus overweight). In both sexes, the presence of metabolic factors in the aggregate did not predict subsequent occurrence of cancer as a whole. By site, a significant increase in risk was observed for liver cancer in men (≧3 factors: HR, 1.73; 95% CI, 1.03–2.91; and, ≧2 additional factors, plus overweight: 1.99, 1.11–3.58), and pancreatic cancer in women (≧2 additional factors, plus overweight: 1.99, 1.00–3.96). For other sites, positive associations were observed only for specific metabolic factors, namely, hypertriglyceridemia and colon cancer in men (1.71, 1.11–2.62), and obesity and breast cancer in women (1.75, 1.21–2.55). Metabolic factors in the aggregate appear to have little impact on overall cancer risk in the Japanese population, although the association between specific components and specific cancers suggests an etiologic link.

## CONCLUSION

We estimated the impact of major risk factors, namely tobacco smoking, alcohol drinking, BMI, history of diabetes, physical activity, and metabolic factors and their aggregates, on overall cancer risk among a Japanese population. Tobacco smoking and heavy alcohol drinking were significantly positively associated with overall cancer risk, and total physical activity was significantly inversely associated with overall cancer risk. In addition, although participants with a history of DM appear to be at increased overall risk of cancer, BMI and metabolic factors in the aggregate had little impact on overall cancer risk in this population.

## APPENDIX

Members of the Japan Public Health Center-based Prospective Study (JPHC Study, principal investigator: S. Tsugane) Group are: S. Tsugane, M. Inoue, T. Sobue, and T. Hanaoka, National Cancer Center, Tokyo; J. Ogata, S. Baba, T. Mannami, A. Okayama, and Y. Kokubo, National Cardiovascular Center, Osaka; K. Miyakawa, F. Saito, A. Koizumi, Y. Sano, I. Hashimoto, T. Ikuta and Y. Tanaba, Iwate Prefectural Ninohe Public Health Center, Iwate; Y. Miyajima, N. Suzuki, S. Nagasawa, Y. Furusugi, and N. Nagai, Akita Prefectural Yokote Public Health Center, Akita; H. Sanada, Y. Hatayama, F. Kobayashi, H. Uchino, Y. Shirai, T. Kondo, R. Sasaki, Y. Watanabe, Y. Miyagawa, Y. Kobayashi and M. Machida, Nagano Prefectural Saku Public Health Center, Nagano; Y. Kishimoto, E. Takara, T. Fukuyama, M. Kinjo, M. Irei, and H. Sakiyama, Okinawa Prefectural Chubu Public Health Center, Okinawa; K. Imoto, H. Yazawa, T. Seo, A. Seiko, F. Ito, F. Shoji and R. Saito, Katsushika Public Health Center, Tokyo; A. Murata, K. Minato, K. Motegi, T. Fujieda and S. Yamato, Ibaraki Prefectural Mito Public Health Center, Ibaraki; K. Matsui, T. Abe, M. Katagiri, and M. Suzuki, Niigata Prefectural Kashiwazaki and Nagaoka Public Health Center, Niigata; M. Doi, A. Terao, Y. Ishikawa, and T. Tagami, Kochi Prefectural Chuo-higashi Public Health Center, Kochi; H. Sueta, H. Doi, M. Urata, N. Okamoto, and F. Ide, Nagasaki Prefectural Kamigoto Public Health Center, Nagasaki; H. Sakiyama, N. Onga, H. Takaesu, and M. Uehara, Okinawa Prefectural Miyako Public Health Center, Okinawa; F. Horii, I. Asano, H. Yamaguchi, K. Aoki, S. Maruyama, M. Ichii, and M. Takano, Osaka Prefectural Suita Public Health Center, Osaka; Y. Tsubono, Tohoku University, Miyagi; K. Suzuki, Research Institute for Brain and Blood Vessels Akita, Akita; Y. Honda, K. Yamagishi, S. Sakurai and N. Tsuchiya, Tsukuba University, Ibaraki; M. Kabuto, National Institute for Environmental Studies, Ibaraki; M. Yamaguchi, Y. Matsumura, S. Sasaki, and S. Watanabe, National Institute of Health and Nutrition, Tokyo; M. Akabane, Tokyo University of Agriculture, Tokyo; T. Kadowaki, Tokyo University, Tokyo; M. Noda and T. Mizoue, International Medical Center of Japan, Tokyo; Y. Kawaguchi, Tokyo Medical and Dental University, Tokyo; Y. Takashima and Y. Yoshida, Kyorin University, Tokyo; K. Nakamura, Niigata University, Niigata; S. Matsushima and S. Natsukawa, Saku General Hospital, Nagano; H. Shimizu, Sakihae Institute, Gifu; H. Sugimura, Hamamatsu University, Shizuoka; S. Tominaga, Aichi Cancer Center Research Institute, Aichi; H. Iso, Osaka University, Osaka; M. Iida, W. Ajiki, and A. Ioka, Osaka Medical Center for Cancer and Cardiovascular Disease, Osaka; S. Sato, Chiba Prefectural Institute of Public Health, Chiba; E. Maruyama, Kobe University, Hyogo; M. Konishi, K. Okada, and I. Saito, Ehime University, Ehime; N. Yasuda, Kochi University, Kochi; S. Kono, Kyushu University, Fukuoka.
